# Bridging paradigms and shaping the future of resectable gastric cancer: a narrative review of the post-MATTERHORN era

**DOI:** 10.1093/gastro/goag010

**Published:** 2026-03-09

**Authors:** Dai Okemoto, Izuma Nakayama, Kohei Shitara

**Affiliations:** Department of Gastroenterology and Gastrointestinal Oncology, National Cancer Center Hospital East, Kashiwa, Chiba 277-8577, Japan; Department of Gastroenterology and Gastrointestinal Oncology, National Cancer Center Hospital East, Kashiwa, Chiba 277-8577, Japan; Department of Gastroenterology and Gastrointestinal Oncology, National Cancer Center Hospital East, Kashiwa, Chiba 277-8577, Japan

**Keywords:** perioperative chemotherapy, neoadjuvant chemotherapy, locally advanced gastric cancer, locally advanced gastroesophageal cancer, immunotherapy, targeted therapy

## Abstract

Gastric or gastroesophageal junction cancer (GC/GEJC) ranks as the fifth leading cause of cancer-related mortality worldwide. Curative-intent surgery remains the cornerstone of treatment for locally advanced GC/GEJC. However, a substantial proportion of patients still experience disease recurrence even after complete resection. Based on the pivotal results of the FLOT4 and ESOPEC trials, perioperative FLOT (fluorouracil, leucovorin, oxaliplatin, and docetaxel) has been established as the standard of care for patients with locally advanced upper gastrointestinal tract adenocarcinoma, including GC/GEJC, in Western countries, whereas surgery followed by adjuvant chemotherapy remains the standard in Asia. The positive findings from the PRODIGY and RESOLVE trials have further supported neoadjuvant and perioperative strategies in Asia, fostering a trend toward global harmonization. Following the first global collaboration in KEYNOTE-585, the randomized phase III MATTERHORN trial represented a major milestone by demonstrating, for the first time, a significant improvement in event-free survival and overall survival with the addition of durvalumab to perioperative FLOT. This provided the first global phase III evidence supporting the integration of immune checkpoint inhibitors with perioperative chemotherapy in GC/GEJC. In this review, we spotlight the paradigm shift in perioperative treatment and address challenges associated with implementing FLOT plus durvalumab in daily practice. We also discuss future therapeutic directions, including molecularly targeted therapies and novel multimodal approaches. The MATTERHORN trial has set the global stage for advancing the management of resectable GC/GEJC, heralding the beginning of a new era.

## Introduction

Gastric cancer is the fifth leading cause of cancer mortality and the fifth most common malignancy worldwide [1]. Surgical resection remains the cornerstone of curative treatment and long-term survival in locally advanced gastric or gastroesophageal junction cancer (GC/GEJC). However, high postoperative recurrence and molecular heterogeneity make management challenging [2]. To address these challenges, perioperative therapy was introduced to improve resectability and reduce recurrence risk [3–5].

Although systemic therapies for metastatic GC/GEJC have advanced globally, the East and West remain divergent in their approaches to therapeutic developments for locally advanced disease [1].

In Western countries, perioperative chemotherapy has been the standard for locally advanced GC/GEJC [4, 5]. The randomized phase III FLOT4 trial established the FLOT regimen (5-fluorouracil, leucovorin, oxaliplatin, and docetaxel) as the standard of care, which is now widely adopted in Western countries [3]. Furthermore, the randomized phase III ESOPEC trial extended FLOT to locally advanced esophageal adenocarcinoma [6].

In contrast, gastrectomy followed by adjuvant chemotherapy has been standard in Asia [7–12]. However, recent studies such as PRODIGY and RESOLVE support perioperative chemotherapy [13, 14], demonstrating the efficacy of docetaxel, oxaliplatin, and S-1 (DOS) and S-1 and oxaliplatin (SOX) therapy. Based on these findings, the ESMO Pan-Asia guidelines recommend perioperative therapy for cT4N+ or bulky nodal disease [15], reflecting a paradigm shift.

Against this background, the KEYNOTE-585 trial—the first global phase III study in this setting—was initiated [16], and positive findings of MATTERHORN trial established FLOT plus durvalumab, an anti–PD-L1 antibody, as a new global perioperative standard (D-FLOT) [17].

To further enhance treatment outcomes, a number of clinical trials are evaluating targeted therapies such as HER2- and VEGF-directed agents, and radiotherapy [18–22].

**Figure 1:**
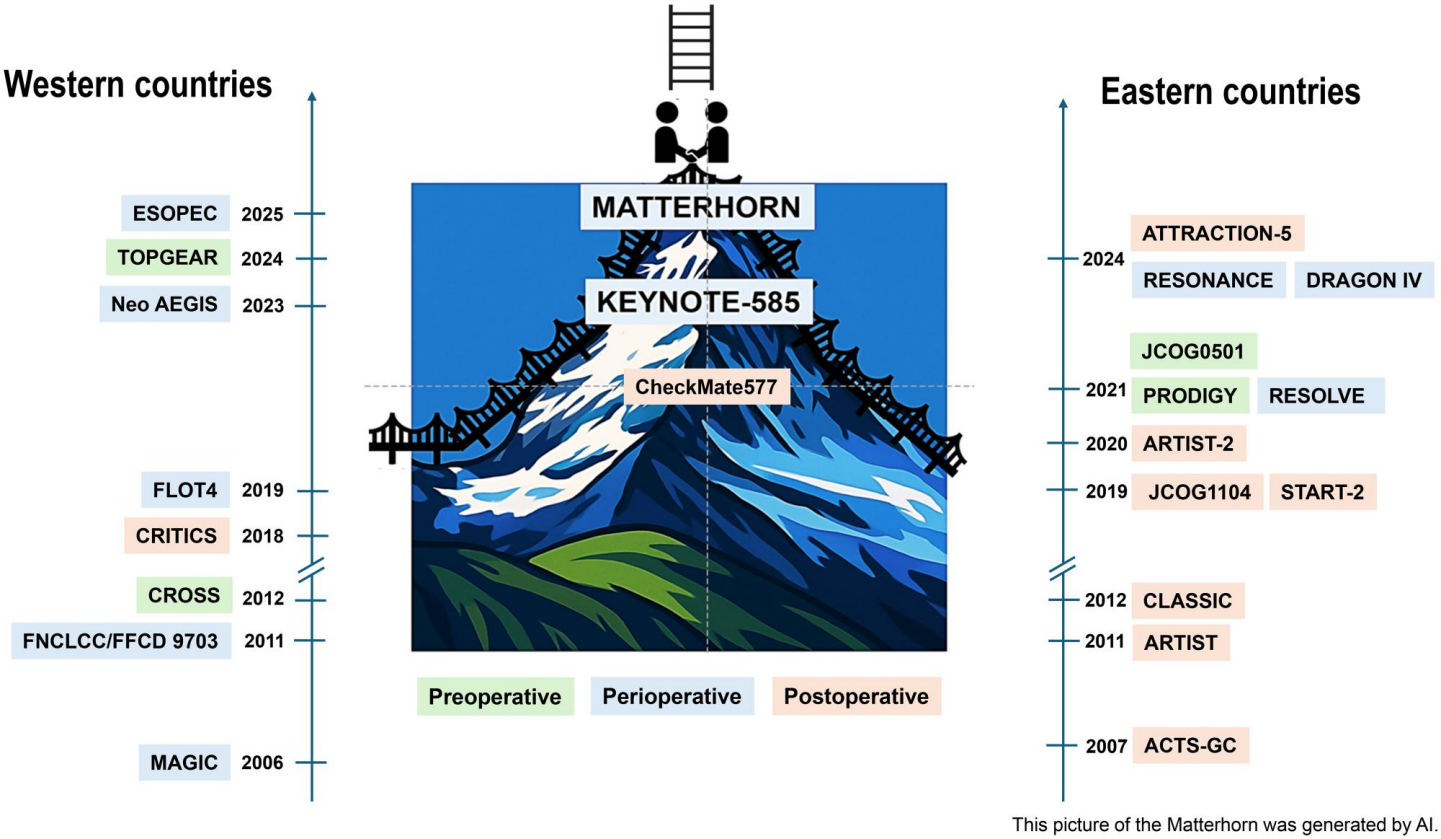
Representative pivotal perioperative trials in gastric and esophagogastric cancer, highlighting differences between Western and Eastern countries and global harmonization of perioperative treatment. This picture of the Matterhorn was generated by AI.

This review provides an overview of the evolving landscape of perioperative treatment for locally advanced resectable GC/GEJC, focusing on pivotal trials (summarized in Fig. 1 and Tables 1–3), biomarkers, and novel strategies.

**Table 1:** Pivotal phase III trials of perioperative treatment for resectable esophagogastric cancer in Western countries

Trial	Phase	Location	*n*	Regimens	Primary endpoint	Outcomes	Result
MAGIC	III	G/GEJ/LE	503	Perioperative ECF vs surgery alone	OS	5Y-OS 36% vs 23%, HR = 0.75, *P *= 0.009	Positive
FNCLCC/FFCD 9703	III	G/GEJ/LE	224	Perioperative FP vs surgery alone	OS	5Y-OS 38% vs 24%, HR = 0.69, *P *= 0.02	Positive
CROSS	III	E/GEJ	368	NAC-CP + RT vs surgery alone	OS	Median OS 49 months vs 24 months, HR = 0.66, *P *= 0.003	Positive
CRITICS	III	G/GEJ	788	Adjuvant ECX/EOX + RT vs adjuvant ECX/EOX	OS	Median OS 43 months vs 37 months, HR = 1.01, *P *= 0.90	Negative
FLOT4	II/III	G/GEJ	716	Perioperative FLOT vs perioperative ECF/ECX	OS	5Y-OS 45% vs 36%, HR = 0.77, *P *= 0.012	Positive
Neo-AEGIS	III	E/GEJ	362	NAC-CP + RT vs perioperative ECF/ECX/FLOT	OS	Median OS 49 months vs 48 months, HR = 1.03, *P *= 0.82	Negative
TOPGEAR	III	G/GEJ	574	Perioperative ECF/ECX/FLOT + CRT vs perioperative ECF/ECX/FLOT	OS	Median OS 46 months vs 49 months, HR = 1.05	Negative
ESOPEC	III	E/GEJ	438	Perioperative FLOT vs NAC-CP+RT	OS	3Y-OS 57% vs 51%, HR = 0.70, *P *= 0.01	Positive

CP, carboplatin + paclitaxel; CRT, chemoradiotherapy; E/GEJ, esophageal or gastroesophageal junction; ECF, epirubicin + cisplatin + fluorouracil; ECX, epirubicin + cisplatin + capecitabine; EOX, epirubicin + oxaliplatin + capecitabine; FLOT, fluorouracil + leucovorin + oxaliplatin + docetaxel; FP, fluorouracil + cisplatin; G/GEJ, gastric or gastroesophageal junction; HR, hazard ratio; LE, lower esophagus; NAC, neoadjuvant chemotherapy; OS, overall survival; RT, radiotherapy; Y, year.

**Table 2:** Pivotal phase III trials of perioperative treatment for resectable esophagogastric cancer in Asia.

Trial	Phase	Location	*n*	Regimens	Primary endpoint	Outcomes	Result
ACTS-GC	III	G	1059	Adjuvant S-1 vs surgery alone	OS	3Y-OS 80% vs 70%, HR = 0.68, *P *= 0.003	Positive
ARTIST	III	G	458	Adjuvant XP + RT vs adjuvant XP	DFS	3Y-DFS 78% vs 74%, *P *= 0.086	Negative
CLASSIC	III	G	1035	Adjuvant CapeOX vs surgery alone	3Y-DFS	3Y-DFS 74% vs 59%, HR = 0.56, *P *< 0.0001	Positive
JCOG1104	III	G	590	Adjuvant S-1 (4 cycles) vs adjuvant S-1 (8 cycles)	RFS	3Y-RFS 90% vs 93%, HR = 1.84	Negative
START-2 (JACCRO GC-07)	III	G	915	Adjuvant DS vs adjuvant S-1	3Y-RFS	3Y-RFS 66% vs 50%, HR = 0.63, *P *< 0.001	Positive
ARTIST-2	III	G	546	Adjuvant SOX + RT vs adjuvant S-1	3Y-DFS	3Y-DFS 73% vs 65%, HR = 0.72, *P *= 0.07	Negative
PRODIGY	III	G/GEJ	530	NAC-DOS + adjuvant S-1 vs adjuvant S-1	PFS	3Y-PFS 66% vs 60%, HR = 0.70, *P *= 0.023	Positive
RESOLVE	III	G/GEJ	1094	Perioperative SOX vs adjuvant CapeOX	3Y-DFS	3Y-DFS 59% vs 51%, HR = 0.77, *P *= 0.028	Positive
JCOG0501	III	G	316	NAC-SP + adjuvant S-1 vs adjuvant S-1	OS	3Y-OS 62% vs 61%, HR = 0.92, *P *= 0.280	Negative
RESONANCE	III	G/GEJ	772	Perioperative SOX vs adjuvant SOX	3Y-DFS	3Y-DFS 62% vs 54%, HR = 0.76, *P *= 0.019	Positive
ATTRACTION-5	III	G/GEJ	755	Adjuvant SOX + nivolumab vs adjuvant SOX	RFS	3Y-RFS 68% vs 65%, HR = 0.90, *P *= 0.44	Negative
DRAGON-IV	II/III	G/GEJ	360 (180 + 180)	Perioperative SOX + camrelizumab + rivoceranib vs perioperative SOX	pCR, EFS	pCR 18% vs 5.0% (*P *< 0.0001); EFS not yet available	Ongoing

CapeOX, capecitabine + oxaliplatin; DFS, disease-free survival; DOS, docetaxel + oxaliplatin + S-1; DS, docetaxel + S-1; EFS, event-free survival; G, gastric; G/GEJ, gastric or gastroesophageal junction; HR, hazard ratio; NAC, neoadjuvant chemotherapy; OS, overall survival; pCR, pathological complete response; PFS, progression-free survival; RFS, recurrence-free survival; RT, radiotherapy; SP, S-1 + cisplatin; S-1, oral fluoropyrimidine derivative; SOX, S-1 + oxaliplatin; XP, capecitabine + cisplatin; Y, year.

**Table 3:** Global pivotal phase III trials of perioperative treatment for resectable esophagogastric cancer

Trial	Phase	Location	*n*	Regimens	Primary endpoint	Outcomes	Result
CheckMate577	III	E/GEJ	794	NAC-CP + RT→ adjuvant nivolumab vs NAC-CP + RT	DFS	Median DFS 22 months vs 11 months, HR = 0.69, *P *< 0.001	Positive
KEYNOTE-585	III	G/GEJ	1007	Perioperative FP/XP/FLOT + pembrolizumab vs perioperative FP/XP/FLOT	pCR, EFS, OS	pCR 13% vs 2% (*P *< 0.00001); median EFS 44 months vs 26 months, HR = 0.81, P = 0.0198; median OS 72 months vs 56 months, HR = 0.86	Negative
MATTERHORN	III	G/GEJ	948	Perioperative FLOT + durvalumab vs perioperative FLOT	EFS	24-month-EFS 67% vs 59%, HR = 0.71, P < 0.001	Positive

CP, carboplatin + paclitaxel; DFS, disease-free survival; EFS, event-free survival; E/GEJ, esophageal or gastroesophageal junction; FLOT, fluorouracil + leucovorin + oxaliplatin + docetaxel; FP, fluorouracil + cisplatin; G/GEJ, gastric or gastroesophageal junction; HR, hazard ratio; NAC, neoadjuvant chemotherapy; OS, overall survival; pCR, pathological complete response; RT, radiotherapy; XP, capecitabine + cisplatin; Y, year.

## The evolution toward a globally accepted standard of care

### Development of evidence in Western countries

Based on the MAGIC, FNCLCC/FFCD, and FLOT4 trials [3–5], perioperative FLOT has become the standard of care in Western countries for resectable GC/GEJC. In Western populations, where adenocarcinoma predominates among esophageal cancers, lower esophageal adenocarcinoma is often included in systemic therapy trials for GC/GEJC. The CROSS trial demonstrated the benefit of preoperative chemoradiotherapy (CRT) using the CROSS regimen—carboplatin and paclitaxel combined with 41.4 Gy of radiotherapy—in esophageal and GEJ cancer, and this approach is also considered a standard [23]. The randomized phase III Neo-AEGIS trial compared the CROSS regimen with perioperative chemotherapy in locally advanced esophageal and GEJ adenocarcinoma. The modified MAGIC regimen (epirubicin plus cisplatin or oxaliplatin plus fluorouracil or capecitabine) was mainly used, while FLOT was given in 15% (*n *= 27). Although terminated early, the 3-year overall survival (OS) was similar—55% with perioperative chemotherapy and 57% with CRT (hazard ratio [HR] 1.03; 95% confidence interval [CI], 0.77–1.38; *P *= 0.82) [20].

Subsequently, the randomized phase III ESOPEC trial compared perioperative FLOT with CROSS regimen in locally advanced resectable esophageal or GEJ adenocarcinoma (cT1–4a cN+, or cT2–4a cN0) [6]. OS was the primary endpoint. At 3 years, OS was 57.4% (95% CI, 50.1–64.0) with FLOT and 50.7% (95% CI, 43.5–57.5) with CROSS regimen, HR 0.70 (95% CI, 0.53–0.92; *P *= 0.01), demonstrating FLOT superiority. A limitation was that non-pCR patients did not receive adjuvant nivolumab, an anti–PD-1 antibody, as the trial preceded CheckMate 577 [24]. However, the final analysis of CheckMate 577 did not demonstrate a statistically significant OS benefit (51.7 vs 35.3 months; HR = 0.85, 95.87% CI, 0.70–1.04; *P *= 0.1064) [25].

The randomized phase III TOPGEAR trial evaluated whether adding preoperative CRT to perioperative chemotherapy improved outcomes in locally advanced resectable gastric or gastroesophageal junction (G/GEJ) adenocarcinoma (cT3–4N0 or N+) [21]. Perioperative chemotherapy was MAGIC regimen (67%) or FLOT (33%), while the CRT group additionally received 45 Gy RT with concurrent fluorouracil before surgery. Although the pCR rate was higher with CRT (17% vs 8%), the primary endpoint, OS, was not improved by CRT (median OS: 46 months with CRT vs 49 months with chemotherapy alone; HR = 1.05, 95% CI: 0.83–1.31).

Collectively, perioperative FLOT has been considered standard care for patients with resectable upper gastrointestinal tract adenocarcinoma in Western countries. However, no head-to-head comparison has been conducted between perioperative FLOT and neoadjuvant CRT followed by postoperative nivolumab for non-pCR patients. Further studies are warranted to determine the optimal dose and timing of radiation therapy in resectable esophageal and GEJ adenocarcinoma.

### Development of evidence in Eastern countries

Based on ACTS-GC, START-2, CLASSIC, and ARTIST-2 trials [7–10], surgery followed by adjuvant chemotherapy is the standard for resectable locally advanced GC. However, due to the challenges of intensifying postoperative treatment, the paradigm in Asia has been shifting toward preoperative or perioperative approach [13, 14, 26, 27].

In Japan, the phase III JCOG0501 trial of neoadjuvant two cycles of S-1 plus cisplatin followed by surgery and adjuvant S-1 did not improve OS compared with upfront surgery plus adjuvant S-1 for scirrhous-type GC [26]. The PRODIGY trial, a randomized phase III study conducted primarily in Korea, assessed neoadjuvant DOS vs upfront surgery plus adjuvant S-1 in patients with cT2–3N+ or T4anyN gastric cancer [13]. This study met its primary endpoint, demonstrating a statistically significant improvement in progression-free survival. Additionally, the final assessment revealed a favorable OS in preoperative DOS arm (adjusted HR = 0.72, 95% CI: 0.54–0.96).

The RESOLVE trial, a randomized phase III study in China, evaluated perioperative SOX and adjuvant SOX compared with adjuvant capecitabine and oxaliplatin (CAPOX) in patients with cT4aN+ or cT4b GC/GEJC [14]. This study met its primary endpoint, demonstrating a statistically significant improvement in disease-free survival. In an updated analysis, 5-year OS was higher with perioperative SOX (60.0%) than with adjuvant CAPOX (52.1%) (HR = 0.79; 95% CI: 0.62–1.00) [28]. These results have helped align the Asian approach closer to the Western standard. Updated ESMO Pan-Asia Guidelines Adaptation 2024 now recommend perioperative/preoperative chemotherapy cT4N+ or bulky nodal disease [15].

### Building evidence through global collaboration

The randomized phase III KEYNOTE-585 trial achieved the first global collaboration for perioperative treatment, which assessed the efficacy of adding pembrolizumab, an anti–PD-1 antibody, to perioperative chemotherapy for patients with cT ≥3 and/or N-positive, M0 GC/GEJC [16]. While pembrolizumab plus chemotherapy yielded a higher pCR rate (12.9%) compared with chemotherapy alone (2.0%), this trial did not demonstrate a statistically significant event-free survival (EFS) benefit. Although the median EFS was longer with pembrolizumab plus chemotherapy (44.4 vs 25.3 months; HR = 0.81; 95% CI: 0.67–0.98; *P *= 0.0198), it did not meet the pre-specified statistical significance threshold (*P* = 0.0178) for this co-primary endpoint. At final analysis, median OS was 71.8 months with pembrolizumab plus chemotherapy vs 55.7 months with chemotherapy alone (HR = 0.86; 95% CI: 0.71–1.06). The trial’s complex design and predominant use of cisplatin-based doublets may have limited benefit. The possible lack of immunogenic cell death induction by cisplatin, in contrast to oxaliplatin, may have contributed to the observed results [29].

The subsequent global phase III MATTERHORN trial evaluated durvalumab plus FLOT in patients with stage II–IVa resectable GC/GEJC [17]. Treatment consists of two cycles of neoadjuvant therapy, each comprising durvalumab or placebo on day 1 and FLOT on days 1 and 15 of a 4-week cycle, followed by surgery. After surgery, patients receive two additional cycles of adjuvant FLOT with durvalumab or placebo, followed by durvalumab or placebo monotherapy every four weeks for up to 10 cycles. The pCR rate, a key secondary endpoint, showed a statistically significant improvement, with an incidence of 19% in the D-FLOT arm compared with 7% in the FLOT arm (*P *< 0.00001). A statistically significant improvement in the primary endpoint, EFS, was observed in the D-FLOT arm compared with the FLOT arm (HR 0.71, 95% CI 0.58–0.86; *P *< 0.001), with 24-month EFS rates of 67% vs 59%, respectively. The EFS benefit was consistent across almost prespecified subgroups. Furthermore, D-FLOT arm significantly improved OS compared with FLOT arm (HR 0.78, 95% CI 0.63–0.96; *P *= 0.021), with 36-month OS rates of 68.6% vs 61.9% [30]. The OS benefit was generally consistent across clinically relevant subgroups, including PD-L1 expression levels and geographic regions. Importantly, treatment completion rates were comparable between arms, and Grade ≥3 AEs occurred at similar frequencies (60% vs 59%), while grade ≥3 immune-related events remained manageable (7% vs 4%).

Following the results of the MATTERHORN trial, D-FLOT has emerged as the first and only globally established perioperative regimen for locally advanced resectable GC/GEJC, highlighting the potential immune checkpoint inhibitors (ICIs) in preoperative setting.

## Clinical implementation of D-FLOT: evidence gaps between clinical trial and clinical practice

Following the MATTERHORN trial, D-FLOT is anticipated to become the cornerstone of perioperative treatment for GC/GEJC. However, several aspects remain beyond its evidence.

According to the ESMO living guideline, perioperative chemotherapy is standard for cTMN Stage Ib–III except cT1N0M0 GC/GEJC. With approval of durvalumab, D-FLOT is expected to largely replace FLOT in real-world practice in Western populations. Unlike the preceding phase II/III FLOT4 trial, MATTERHORN excluded patients with cT2N0M0 or cT1N1M0 disease, creating an evidence gap for this subgroup. The investigator-initiated, multicenter, randomized, unblinded, controlled phase II/III study, the DANTE trial, is evaluating perioperative atezolizumab, an anti–PD-L1 antibody, plus FLOT vs FLOT alone in resectable GC/GEJC with stage ≥T2 or node-positive disease [31]. The interim analysis showed improved pathological response with the addition of atezolizumab. Further results will clarify broader applicability of ICI with FLOT in resectable GC/GEJC.

In MATTERHORN, the median age was 62 years; 59% were younger than 65, and only 8% were aged 75 or older. The majority of patients with GC/GEJC—nearly two-thirds—are over the age of 65 in the world [32].

A study evaluating the tolerability of FLOT vs 5-fluorouracil, leucovorin, and oxaliplatin (FLO) in patients aged ≥65 with resectable or unresectable gastric cancer found a significantly higher incidence of grade ≥3 adverse events in the FLOT group (81.9%) compared with the FLO group (38.6%) (*P *< 0.001) [33]. Although there was no statistical significance, patients receiving FLOT tended to have a longer median progression-free survival than those in the FLO group (9.0 vs 7.1 months; *P *= 0.079). FLO did not appear to be a viable alternative to FLOT for patients aged ≥65. Japanese real-world data showed dose reduction in 77.8% of patients aged 75 years and older, compared to 50.0% in those younger than 75 years (*P *= 0.014). In clinical practice, appropriate dose reductions are effective in managing toxicities in elderly patients [34].

MATTERHORN recruited nearly 150 institutions across 20 countries, but over half were European. Asian patients comprised 19%, none were from China, which accounts for approximately 35% of global gastric cancer [35]. Notably, HR for EFS was consistently favorable in Asians (HR, 0.74; 95% CI, 0.44–1.22) as well as non-Asians (HR, 0.70; 95% CI, 0.56–0.87). Furthermore, trials in metastatic GC/GEJC also show no regional differences in ICI benefit [36–38]. There is little controversy regarding the benefit of adding ICIs in Asian patients.

## Open questions regarding D-FLOT regimen

D-FLOT has become the first global consensus regimen for resectable locally advanced GC/GEJC, yet its limitations underscore the need for refinement.

### Is postoperative D-FLOT the optimal approach?

In MATTERHORN, among 474 patients in the D-FLOT arm, 360 (75.9%) initiated postoperative treatment, but only 229 (48.3%) completed all planned adjuvant cycles, and rates were nearly identical in the FLOT arm. Similarly, in FLOT4 trial, completion of both pre- and postoperative FLOT was only 46% [4]. A Japanese retrospective analysis reported 51.6% [34].

In Asia, de-escalation strategies have been adopted according to pathological stage, with patients typically receiving triplet chemotherapy preoperatively, followed by reduced regimens (doublet or monotherapy) postoperatively. In PRODIGY, adjuvant S-1 was given postoperatively in both arms, and completion rate among initiators was relatively high at 83% [13]. Japanese studies have also implemented de-escalation strategies after preoperative triplet therapy followed by surgery [39, 40]. However, the recent single-arm Japanese phase II OGSG1902 trial evaluating perioperative DOS in scirrhous-type GC reported an overall completion rate of only 45.8% [41]. Differences in surgical procedures may contribute to this finding: in the PRODIGY trial, 120 of 222 patients (54.1%) underwent total gastrectomy after preoperative DOS, whereas in the OGSG1902 trial, total gastrectomy was performed in 32 of 47 patients (68.1%). Future strategies should address the difficulty of completing adjuvant chemotherapy after total gastrectomy.

The prospective observational SPACE-FLOT study highlighted differences in the efficacy of adjuvant therapy according to pathological stage [42]. No survival benefit from adjuvant FLOT was observed in pCR patients, suggesting that postoperative FLOT may be omitted in selected cases. Similarly, a retrospective analysis reported a questionable benefit of postoperative FLOT in the ypN-negative subgroup [43]. However, given the substantial imbalances in patient characteristics between adjuvant and non-adjuvant groups, these retrospective findings should be interpreted with caution.

The European randomized phase II VESTIGE trial investigated replacing adjuvant FLOT with nivolumab plus ipilimumab, an anti-CTLA-4 antibody, in patients at high risk of recurrence (ypN+ or R1 resection) after curative surgery [44]. This study was terminated early; median disease-free survival was 20.8 months (95% CI, 15.0–29.9) with FLOT vs 11.4 months (95% CI, 8.4–16.8) with dual ICI, corresponding to a HR of 1.55 (95% CI, 1.07–2.25; one-sided *P *= 0.99). These data indicated worse outcomes in the ICI arm. Thus, FLOT remains standard in the adjuvant setting.

As in other tumors, circulating tumor DNA (ctDNA) assays may enable a more personalized approach and optimize postoperative management in GC/GEJC. However, peritoneum is the main recurrence site, and the limited cell release of tumor into the circulation poses a major challenge for clinical adoption of ctDNA in minimal residual disease detection. Advances in ultra-sensitive, whole genome-based assays may help to overcome this limitation [45].

MATTERHORN confirmed the efficacy of D-FLOT in resectable GC/GEJC, but could not clarify whether benefit derived from preoperative, postoperative, or both. By contrast, the Asian randomized phase III ATTRACTION-5 trial showed no significant benefit from adding nivolumab to adjuvant chemotherapy (S-1 or CAPOX) after curative surgery [46]. Future trials should assess the independent contribution of each treatment component.

### Is FLOT the optimal backbone chemotherapy for ICI-based therapy?

The RESOLVE trial demonstrated the efficacy of perioperative SOX chemotherapy [14]. Several single-arm phase II trials combining ICIs with SOX or CAPOX have demonstrated promising activity, achieving pCR rates of 19%–33% [47–49]. Furthermore, a randomized phase II NEOSUMMITE-01 trial (*n *= 108) evaluated the additive effect of toripalimab into SOX/CAPOX, demonstrating significantly improved pathological response, defined by tumor regression grade (TRG) 0/1 (44% vs 20%; *P *= 0.01) [50]. The addition of toripalimab resulted in a higher pCR rate (14.8%–22.2% with toripalimab vs 7.4% without; *P *= 0.030). A prospective comparison between neoadjuvant FLOT and SOX demonstrated lower pathological response (TRG 1a/1b) in FLOT arm (20.0%) than that in SOX (32.4%). In addition, there was no significant difference in hematological and gastrointestinal toxicities between two arms. However, as this was an exploratory analysis (*n *= 74) conducted without a statistical power calculation, the findings should be interpreted with caution [51].

DOS is another promising triplet backbone, particularly in Asian countries, as an alternative to FLOT. A single-arm phase II trial evaluating DOS plus durvalumab demonstrated a pCR rate of 30% [52], suggesting that DOS could serve as a viable platform for future combination strategies. In China, a randomized phase II/III RESOLVE-2 trial (NCT03691454) is currently evaluating perioperative 8 cycles of DOS vs 6 cycles of SOX in patients with cStage III/IVa GC/GEJC (AJCC 8th edition). Meanwhile, prospective studies comparing the safety and efficacy of preoperative FLOT and DOS are currently underway in Japan [40, 53].

The global adoption of S-1-based regimens remains limited due to interethnic variability in metabolic activation and tolerability. Substituting S-1 with capecitabine could enable broader global applicability of a triplet regimen that incorporate an oral fluoropyrimidine. The phase II PANDA trial adopted a DOC regimen (docetaxel 50 mg/m^2^, oxaliplatin 100 mg/m^2^, and capecitabine 850 mg/m^2^) as the backbone. Neoadjuvant treatment with one cycle of atezolizumab followed by four cycles of DOC achieved a major pathological response (MPR) rate of 70% (95% CI: 46%–88%) and a pCR rate of 45% (95% CI: 23%–68%) among 20 patients [54]. However, it should be noted that this study was primarily designed to evaluate safety and included a very limited sample size.

Given the negative outcome of KEYNOTE-585 with a cisplatin-based regimen, the immunogenic cell death-inducing properties of oxaliplatin suggest that oxaliplatin-based chemotherapy may serve as a more suitable backbone for immunotherapy combinations. While docetaxel does not induce DNA damage and thus has limited immunogenic cell death-inducing ability, preclinical study has demonstrated that docetaxel can still promote immunogenic modulation and augment cytotoxic T lymphocyte-mediated antitumor activity [55]. Therefore, combining oxaliplatin and docetaxel may provide synergistic immunogenic benefits.

Since FLOT is not universally applicable particularly for elderly or vulnerable patients, optimization of backbone chemotherapy for anti-PD-1/PD-L1 therapy remains an important challenge. Several studies have demonstrated promising feasibility and antitumor activity with alternative chemotherapy regimens combined with ICIs. However, no large-scale randomized phase III trial has yet confirmed superior efficacy over FLOT-based approaches. Furthermore, a large-scale phase III trial to identify alternative backbone regimens is unlikely to be feasible. In this context, the MATTERHORN study currently provides the only robust global evidence supporting perioperative chemo-immunotherapy in resectable, locally advanced GC/GEJC. As already implemented in clinical practice with FLOT, dose-modification for high-risk patients may represent a more pragmatic strategy moving forward.

### Unresolved questions regarding biomarker-based benefit

In the metastatic setting, GC/GEJC with MSI-H/dMMR represents an immunogenic subset, and preferential use of ICIs with chemotherapy is strongly recommended. Although PD-L1 remains a suboptimal biomarker, both NCCN and ESMO guidelines recommend ICIs for metastatic disease in patients with combined positive score (CPS) ≥1 [56, 57].

The interim analysis of the DANTE trial was consistent with these findings. In resectable MSI-H GC/GEJC, the pCR rate reached 50% with FLOT plus atezolizumab. A stepwise increase in pCR was observed with higher CPS: 23% for CPS ≥1, 25% for CPS ≥5, and 30% for CPS ≥10 [31]. In KEYNOTE-585, pembrolizumab produced a larger incremental pCR benefit in CPS ≥1 (+12.1%) than in CPS <1 (+4.2%), and an even greater difference between MSI-H (+37.1%) and non-MSI-H subgroups. Subgroup analyses indicated that EFS benefit occurred across subgroups, with greater improvement in CPS ≥1 and particularly MSI-H tumors. Median EFS was numerically longer in CPS ≥1 (57.7 months) and MSI-H (not reached) than in CPS <1 (33.9 months) or non-MSI-H (35.3 months) [16].

In MATTERHORN, the HR for EFS was 0.70 (95% CI, 0.57–0.87) in PD-L1 tumor area positivity (TAP) ≥1, vs 0.77 (95% CI, 0.40–1.46) in TAP <1 [17]. A similar trend was observed for OS, with an HR of 0.79 (95% CI, 0.63–0.99) in TAP ≥1 and 0.79 (95% CI, 0.41–1.50) in TAP <1 [30], suggesting that the survival benefit of perioperative durvalumab was not restricted to PD-L1–expressing tumors. Nevertheless, NCCN recommends perioperative durvalumab for PD-L1–positive disease (CPS/TAP ≥1), consistent with metastatic indications. However, MATTERHORN was a positive all-comer study, and TAP <1 tumors comprised only about 10% of patients. Therefore, it is premature to conclude that durvalumab lacks efficacy in TAP <1 tumors, and further research is warranted to clarify its potential role in this subgroup.

A randomized phase III trial (NCT04139135) conducted in China evaluated HLX10, an anti-PD-1 antibody, with SOX chemotherapy for PD-L1–positive GC/GEJC (cT3–4N+).

## Developing the novel approaches beyond D-FLOT

In spite of the emergence of perioperative D-FLOT, more than 30% of patients’ experience recurrence within 2 years. The success of biomarker-driven approaches in metastatic GC/GEJC is expected to shape strategies for locally advanced disease.

### HER2-positive GC/GEJC

Two single-arm phase II trials (NEOHX and HERFLOT) and the randomized Japanese JCOG1301C (Trigger) trial suggested activity for adding trastuzumab, an anti-HER2 antibody, to perioperative chemotherapy, but none completed due to poor accrual [58–60]. The randomized PETRARCA and INNOVATION trials tested trastuzumab with or without pertuzumab, an anti-HER2 antibody, showing higher pCR rate but also excess toxicity and no OS benefit [18, 61]. Collectively, these findings indicated dual HER2 blockade is too toxic in the perioperative setting and emphasize the challenges of conducting large-scale trials, underscoring the need for international collaboration.

Given the success of KEYNOTE-811 in the metastatic setting [38], HER2-targeted and ICI combinations are being explored perioperatively. A phase II trial (NCT03950271) tested neoadjuvant camrelizumab, anti-PD-1 antibody, plus trastuzumab and CAPOX in HER2-positive locally advanced GC/GEJC, achieving a pCR rate of 21.7% [62]. Another phase II trial (ChiCTR2200058732) of perioperative sintilimab, an anti-PD-1 antibody, plus trastuzumab and SOX, demonstrated a pCR rate of 50%. Furthermore, a phase II trial (NCT04661150) evaluated the addition of atezolizumab to trastuzumab plus CAPOX vs CAPOX alone, meeting its primary endpoint with a higher pCR rate (38.1% vs 14.3%) [63].

A phase II multicenter trial (NCT04819971) of perioperative tislelizumab, an anti-PD-1 antibody, in combination with trastuzumab, and DOS, reported a pCR rate of 47.1% [64, 65]. Recently a phase II PHERFLOT trial evaluating perioperative FLOT plus pembrolizumab and trastuzumab demonstrated a pCR rate of 48.4%, thereby meeting the co-primary endpoint [66].

Antibody-drug conjugates are also progressing. The single-arm phase II EPOC2003 trial (NCT05034887) evaluated neoadjuvant trastuzumab deruxtecan, an HER2-directed antibody-drug conjugate, as monotherapy in resectable HER2-positive GC/GEJC, but showed modest efficacy, with an MPR rate of 14.8% and a pCR rate of 3.7%, both below threshold [67]. An expansion cohort combining trastuzumab deruxtecan with durvalumab and capecitabine is currently ongoing. Disitamab vedotin is another HER2-directed antibody–drug conjugate, composed of hertuzumab—a novel antibody recognizing a distinct epitope in HER2 subdomain IV—linked to the cytotoxic payload monomethyl auristatin E (vedotin), which disrupts tubulin polymerization [68]. A phase II single-arm study (ChiCTR2300075446) evaluated neoadjuvant disitamab vedotin with camrelizumab and S-1 in HER2-overexpressing, resectable GC/GEJC (cT3–4aN1–3M0), reporting encouraging activity with an MPR rate of 50% and a pCR rate of 31%. Median disease-free survival and OS have not yet reached [69]. A multicenter randomized phase II trial (NCT06155383) is ongoing to compare perioperative toripalimab plus disitamab vedotin and CAPOX vs disitamab vedotin plus toripalimab vs CAPOX [70].

Collectively, these experiences indicate that advancing HER2-targeted strategies will rely more on optimized trial design and global collaboration than on simply developing new targeted agents.

### MSI-H/dMMR

The IMHOTEP trial assessed one or two courses of neoadjuvant pembrolizumab monotherapy across MSI-H/dMMR tumors, including 39 esophagogastric cancers [71]. Among 29 surgical patients, the pCR rate was only 19.4%. Meanwhile, in a recent multicohort phase II study of neoadjuvant dostarlimab administered for nine cycles in patients with dMMR solid tumors, 8 of 15 patients with gastric cancer (57%) achieved a clinical complete response [72].

The phase II NEONIPIGA trial in France evaluated neoadjuvant nivolumab plus ipilimumab in cT2–4NanyM0 MSI-H/dMMR GC/GEJC [73]. Among 29 surgical patients, all achieved R0 resection and 58.6% achieved pCR, while three patients declined surgery after cCR, suggesting the feasibility of non-operative management. The phase II INFINITY trial tested tremelimumab, an anti-CTLA-4 antibody, plus durvalumab in locally advanced MSI-H/dMMR GC/GEJC (≥cT2 and/or N+) [74]. In Cohort 1, pCR and MPR rates reached 60% and 80%, with all responders showing negative ctDNA before surgery. Importantly, efficacy was lower in cT4 disease, low tumor mutation burden, or heterogeneous pMMR/dMMR tumors (pCR 17% vs 89% in non-cT4, *P *= 0.011). Cohort 2 evaluated non-operative management: 76% (13 of 17) achieved cCR and deferred surgery, with only one local regrowth at 11.5 months requiring salvage surgery. However, this cohort closed early before reaching the primary endpoint of 2-year cCR without salvage surgery.

The NICE trial (NCT04744649) further supported perioperative ICI, with toripalimab plus CAPOX producing an MPR rate of 93.3% and a pCR rate of 80% in MSI-H/dMMR GC/GEJC [75]. Similarly, in the DANTE trial, the pCR rate was higher with FLOT plus atezolizumab vs FLOT alone in MSI-H/dMMR (50% vs 27%).

Taken together, these studies highlight the pronounced sensitivity of MSI-H/dMMR GC/GEJC to immunotherapy. Dual ICI regimens appear more effective than monotherapy, and non-operative management has emerged as a potential strategy. However, the optimal regimen, duration, and chemotherapy combination remain unresolved, particularly for subgroups such as cT4, low tumor mutation burden, or heterogeneous dMMR.

### Anti-VEGF

Angiogenesis drives cancer progression by supplying oxygen and nutrients through new vessel formation. Vascular endothelial growth factor (VEGF)-driven vasculature is abnormal and leaky, fostering a hypoxic tumor microenvironment that promotes immunosuppression via regulatory T cells, myeloid-derived suppressor cells, and inhibitory cytokines such as transforming growth factor-β and interleukin-10 [76]. Anti-VEGF therapies normalize vasculature and attenuate these mechanisms, thereby enhancing T-cell infiltration and reprogramming the tumor microenvironment for antitumor immunity [77]. These effects may synergize with ICIs, as demonstrated in phase III trials of VEGF inhibitors plus ICIs in hepatocellular and renal carcinomas [78, 79].

Building on this rationale, several perioperative studies tested VEGF inhibition with ICIs in GC. The DRAGON IV/CAP 05 trial, a randomized phase II/III study in China, tested SOX plus rivoceranib, a vascular endothelial growth factor receptor (VEGFR)-2 tyrosine kinase inhibitor, and camrelizumab reporting a higher pCR rate than SOX alone (18.3% vs 5.0%), meeting one co-primary endpoint, while EFS is pending [19]. Another randomized phase II trial (NCT04195828) evaluated camrelizumab plus rivoceranib with nab-paclitaxel and S-1 vs chemotherapy, achieving a significantly higher MPR rate (33.3% vs 17.0%, *P *= 0.044), although survival results are not yet available [80]. In Japan, the phase II EPOC2001 trial (NCT04745988) assessed lenvatinib, a multikinase inhibitor of VEGFR, fibroblast growth factor receptor, rearranged during transfection, KIT proto-oncogene, receptor tyrosine kinase (KIT), and platelet-derived growth factor receptor α, plus pembrolizumab with FLOT, achieving an MPR rate of 47% and a pCR rate of 22% among 32 patients; survival follow-up is ongoing [81].

However, in the metastatic setting, the phase III LEAP-015 trial failed to demonstrate a significant OS benefit for pembrolizumab plus lenvatinib with chemotherapy over chemotherapy alone in HER2-negative GC/GEJC [82]. Increased toxicities with lenvatinib plus pembrolizumab may have offset potential gains from tumor microenvironment remodeling. This negative result tempers enthusiasm for VEGF-ICI combinations despite encouraging pathological responses in earlier trials. Looking forward, bispecific antibodies targeting VEGF and PD-L1, such as ivonescimab, have shown promising efficacy in metastatic setting, and evaluation in locally advanced disease is anticipated [83].

### Radiotherapy

Subgroup findings suggest potential benefit of preoperative CRT in GEJ adenocarcinoma. In TOPGEAR, patients with GEJ adenocarcinoma showed a trend toward improved OS with CRT (HR 0.78; 95% CI 0.55–1.12) [21]. Similarly, Neo-AEGIS reported a favorable OS trend for CRT over perioperative chemotherapy in AEG type II/III cancers (HR 0.79; 95% CI 0.48–1.31) [20]. Stahl *et al*. suggested an OS advantage with CRT in Siewert type I/II tumors (HR 0.65; 95% CI: 0.42–1.01), although the study was terminated prematurely [84]. Collectively, these data may suggest that preoperative CRT may have a role in selected patients with GEJ adenocarcinoma. The ongoing phase III RACE trial is expected to further clarify this question by comparing perioperative FLOT vs induction FLOT followed by CRT (fluorouracil, oxaliplatin, 45 Gy) prior to surgery in locally advanced, resectable type I–III adenocarcinomas of the gastroesophageal junction [85].

In parallel, combinations of CRT and ICIs have gained momentum. In GC/GEJC, two phase II studies reported pCR rates of 33%–38% [86, 87]. In Japan, the ongoing phase II EPOC2301 trial is investigating a total neoadjuvant therapy strategy for Siewert type I–III GEJ adenocarcinoma (cT2–4, Nany, M0), aiming to evaluate both survival and organ preservation. Patients receive induction FLOT plus pembrolizumab, followed by short-course radiotherapy (25 Gy in 5 fractions), then further FLOT and pembrolizumab. After response assessment, cCR cases may undergo non-operative management, while those with residual disease proceed to surgery. This trial will provide insights into combining immunotherapy and RT for effective perioperative management and organ preservation.

### Others

Building on positive results in the metastatic setting, a multicenter phase II/III trial (NCT05149807) is investigating SHR-1701, a bifunctional fusion protein targeting PD-L1 and transforming growth factor-β, an immunosuppressive cytokine implicated in tumor progression, in combination with neoadjuvant SOX chemotherapy in resectable disease. Lymphocyte-activation gene 3, an inhibitory immune checkpoint receptor expressed on activated T cells, has recently emerged as a novel immunotherapy target [88]. The IMAGINE trial (NCT04062656) is a randomized phase II study testing perioperative nivolumab plus relatlimab, a lymphocyte-activation gene 3 inhibitor, in resectable GC/GEJC.

Claudins (CLDNs) have emerged as novel therapeutic targets. CLDN18.2, selectively expressed on normal gastric mucosa and preserved during malignant transformation [89], has become clinically relevant following the success of the SPOTLIGHT and GLOW trials [90, 91]. The multicohort phase II ILUSTRO trial (NCT03505320) includes a perioperative cohort evaluating FLOT plus zolbetuximab, the first-in-class CLDN18.2-targeting monoclonal antibody, in resectable GC/GEJC. However, zolbetuximab-induced gastritis, manifested as severe nausea, vomiting, and appetite loss during and after the first infusion, represents a unique gastrointestinal toxicity that may complicate its perioperative use.

Cancer vaccines are designed to elicit tumor-specific immune responses, with recent advances in mRNA technology enabling rapid, individualized vaccine development. Another emerging strategy is personalized cancer vaccination. mRNA-4157, a vaccine encoding up to 34 patient-specific neoantigens identified by tumor and normal tissue sequencing, is being investigated in the ongoing phase I/II KEYNOTE-603 trial (Part E3). This trial is evaluating perioperative mRNA-4157 in combination with pembrolizumab and chemotherapy in patients with resectable GC/GEJC (NCT03313778).

## Conclusions

Resectable GC/GEJC has historically been managed differently in Eastern and Western countries. The MATTERHORN study has bridged these paradigms, establishing D-FLOT as the first globally harmonized perioperative regimen. Building on this foundation, the next step will be the worldwide development of more effective treatment strategies.

Integration of targeted agents and novel modalities—such as mRNA vaccines, antibody-drug conjugates, and bispecific antibodies—may provide additional benefit for patients not cured with D-FLOT alone. For patients achieving a CR, organ preservation represents an important future therapeutic goal, with MSI-H/dMMR tumors currently being the most promising candidates. Conversely, some patients may be adequately treated with less intensive approaches, including de-escalation from full FLOT, underscoring the need to refine treatment intensity. To guide such individualized strategies, next-generation ctDNA assays based on whole-genome approaches are anticipated to play a pivotal role in postoperative monitoring and treatment adaptation.

The MATTERHORN trial is not the endpoint but rather the beginning of a new era in the treatment of resectable gastric and GEJ cancer.

## Authors’ contributions

D.O. conceived and carried out the investigation and drafted the original manuscript. I.N. and K.S. supervised the study, and contributed to writing, review, and editing of the manuscript. All authors read and approved the final manuscript.
